# Papillary carcinoma in correlation to thyroidal duct cyst: A case series

**DOI:** 10.22088/cjim.11.1.110

**Published:** 2020

**Authors:** Adele Bahar, Zhila Torabizadeh, Marzieh Movahedi Rad, Zahra Kashi

**Affiliations:** 1 Department of Internal Medicine, Mazandaran University of Medical Sciences, Sari, Iran; 2 Diabetes Research Center, Mazandaran University of Medical Sciences, Sari, Iran; 3 Department of pathology, Mazandaran University of Medical Sciences, Sari, Iran; 4 Gut and liver Research Center, Mazandaran University of Medical Sciences, Sari, Iran

**Keywords:** Thyroglossal duct cyst, Sistrunk, Thyroid carcinoma

## Abstract

**Background:**

Persistent thyroglossal duct leads to a fibrous cyst formation named thyroglossal cyst which is the most common form of congenital cyst and usually located in the midline neck below the hyoid bone. Rarely the thyroglossal cyst is associated with thyroid cancer. Although the Sistrunk procedure is often considered adequate, currently there is no clear consensus on the optimal management of thyroglossal duct cyst especially duct cyst carcinoma. In addition, there is no consensus about concurrent thyroidectomy in patients with thyroglossal cyst duct carcinoma.

**Case presentation:**

In this article, we present four patients with thyroglossal duct cyst and papillary thyroid cancer. Papillary thyroid cancer was located into the thyroglossal duct cyst (thyroglossal duct carcinoma) in two patients and two patients had thyroglossal duct cyst with thyroid carcinoma in thyroid tissue. Cervical lymph nodes were involved in one of the three patients. Sistrunk procedure and total thyroidectomy were performed for all. The thyroid lobes were not involved in patients with thyroid duct cyst carcinoma. Tumor or thyroglossal duct cyst recurrence did not occur in any of the patients in follow-up.

**Conclusion:**

The correlation between thyroglossal cyst and papillary thyroid carcinoma is high. In subjects with thyroglossal duct cyst, in addition to cyst assessment, thyroid gland and neck lymph nodes should be evaluated for malignancy.

Thyroglossal duct cyst (TGDC) is a developmental abnormality of the thyroid gland during the embryonic period. The thyroid gland originates from the base of the tongue and migrates through the thyroglossal tract to its normal anatomic location in the neck. After the migration phase and maturation of the thyroid gland, and thyroglossal tract atrophies. If the canal does not atrophy completely, a thyroglossal duct cyst is formed. Although most patients with TGDC are children or adolescents, up to one-third are aged 20 years or older (1-3). TGDC is present in approximately 7% of the general population (4, 5) . TGDC is the most common congenital mass found at the midline of the neck, however, 10% of TGDC can also be found in the lateral neck (1, 6, 7). Males and females are equally affected. Patients with a TGDC have the ectopic thyroid tissue within the cyst. Generally, duct cysts are benign, but 1% of cases may be malignant (8). Most cases of TGDC carcinoma are diagnosed during the third and fourth decades, and rarely in children less than 14 years of age (9). The first case of the thyroglossal duct cyst papillary carcinoma was reported by Ucherman in 1915. So far, around 260 cases have been described in the literature (10). Ninety percent (90%) of the thyroglossal duct cyst carcinomas are the papillary carcinoma. Other common tumors are squamous cell carcinoma, follicular and hurtle cell carcinoma. Anaplastic thyroid carcinoma is rare and medullary thyroglossal duct cyst carcinoma has not been reported (11). 

The thyroglossal duct carcinoma is diagnosed after the Sistrunk surgery and histopathologic examination in most of the patients (12). The rate of TGDC recurrence is 55.6% with simple cyst excision procedure. The recurrence rate can be reduced to 5.3% with sistrunk procedure (excision of the cyst, the body of the hyoid bone and a core of tissue around the thyroglossal tract) (13). Surgical strategy for TGDC cancer is Sistrunks procedure with or without total thyroidectomy (14). Coexistence of malignant tumor in the thyroid gland has been reported in 27% - 56% of patients with TGDC carcinoma (15, 16). Thyroglossal duct cyst is the most common middle-line anomaly, according to its frequency (7%) and 1% chance of malignancy, it is acceptable to report .Specifically, before making any decision ,the evaluation of a cyst is necessary with fine needle sampling to reject an infection or malignancy.

## Case presentation

**Case one: **A 30-year-old man with a history of hyperthyroidism treated with methimazol starting 36 months ago, was referred for his new complaint about the difficulty in swallowing. Thyroid function test was normal and the patient had no other complaints. There was no history of radiation to the head and neck. On physical examination, there was a soft and mobile 3*2 cm mass in the midline of the neck at the level of the hyoid bone. Neck ultrasonography revealed a 13 mm hypo echoic, solid nodule in the right lobe of thyroid and also a 25 mm hypoechoic nodule with internal echo and abundant echogenic centers in the midline of sub mental region, in favor of the thyroglossal duct cyst. Computed tomography (CT) of the neck revealed a loculated cystic mass approximately 25 mm in diameter in the anterior midline of neck, between the hyoid bone and thyroid cartilage associated with thyrohyoid membrane defect and pre-epiglottic space bulging. Cytological result of sonography guided fine needle aspiration biopsy (FNA) of the thyroid mass was suggestive of the papillary thyroid carcinoma. 

**Figure 1:**
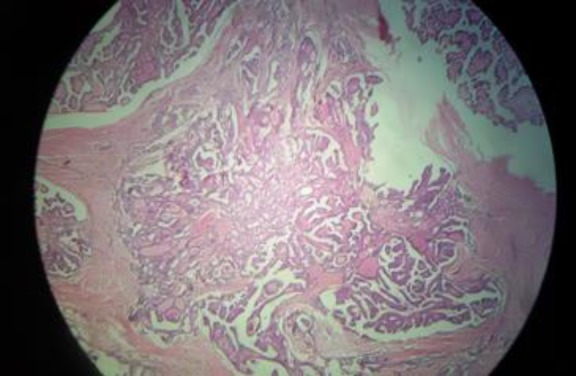
Papillary microcarcinoma

The patient underwent the near total thyroidectomy and Sistrunks procedure for the midline neck mass. The frozen and permanent pathology examination was in favor of papillary microcarcinoma (0.9 cm in diameter) in the right lobe without capsular and vascular invasion and cervical lymph node involvement. The midline neck mass was reported as nonmalignant thyroglosal duct cyst (figure1).

After surgery, he received levothyroxine 800 µg/week without radioiodine therapy. Now after seven years follow-up, the patient is clinically good with acceptable laboratory (suppress thyroglobulin and anti- thyroglobulin) and normal neck ultrasonography without lymph adenopathy and recurrence. 

**Case two: **A 28-year-old woman with a lump in neck midline starting 6 years ago was referred to endocrine clinic. She had no specific complaint except the cosmetic. On physical examination, a firm mobile mass about 4 cm in diameter was touched. Thyroid size was twice as normal without palpable nodule. Her past history was remarkable and thyroid function test was normal.The neck ultrasonography revealed normal right and left thyroid lobes and a 23×16 millimeter solid cystic mass in submental region. The differential diagnosis was a dermoid cyst and complicated thyroglossal duct cyst. Two lymph nodes in the first 9×5 mm in sub mental (level IA) and the second ill-defined border 15 ×8 mm in submandibular region was reported. Computed tomography (CT) was requested for the patient. Spiral contrast neck CT showed a complex cystic mass (4× 4.2 ×2.3 cm) in left para /midline infrahyoid region with extension to the hyoid level.

**Figure 2:**
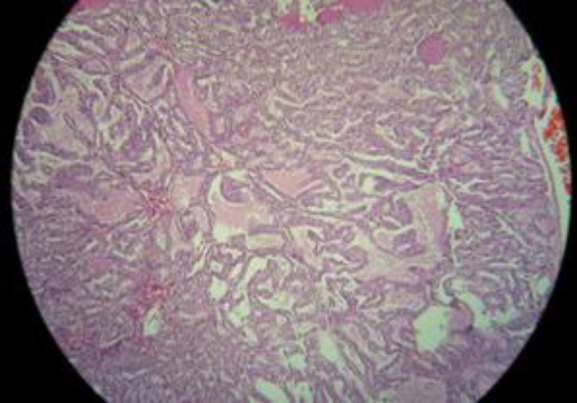
Cystic space containing papillary carcinoma

Sono guided fine needle aspiration biopsy of the neck mass was done. The cytology report showed the small groups and individual follicular cells with mild nuclear grooving, overlapping, anisocytosis and few pseudo inclusion suggested for papillary carcinoma. The patient underwent near total thyroidectomy and the neck mass excision with Sistrunk’s procedure. Pathologic evaluation of the neck mass was in favor of well-differentiated papillary carcinoma, 3 cm in maximum diameter size. Tumor limited to cyst capsule. Lymphatic, blood vessel and perineural invasion were not seen** (**figure 2).

Pathologic evaluation of thyroid right and left lobes revealed the hashimoto's thyroiditis with no malignancy. Neck lymph nodes were free of tumor. The patient received 100 millicuries radioactive iodine after surgery. Right now, after two years, the patient laboratory test and neck ultrasonography are normal with 1000 µg/week levothyroxine therapy.

**Figure 3:**
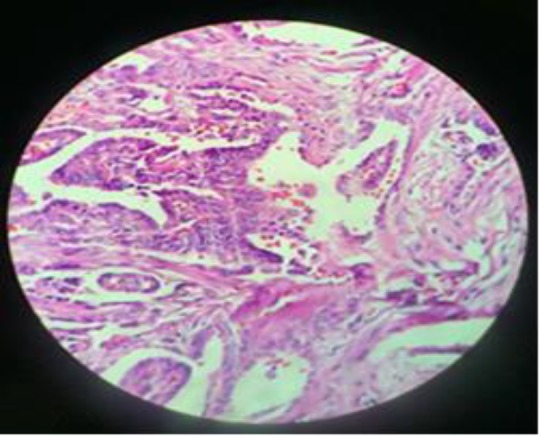
Papillary structures filled the thyroglossal duct cyst

**Case three: **A 43-year old woman had been referred to the endocrine clinic for her diabetes control. At the first visit her thyroid exam was normal and she was euthyroid. After a four-year follow-up she noticed a mass below her chin. An ultrasounography was requested for mass evaluation. Ultrasonic result showed a 33×11 mm cystic mass containing micro calcification at sub mental area in midline but her thyroid was normal, neck lymphadenopathy was not observed. She was advised for the neck mass FNA, but she refused. In control sonography, the mass size became larger (41×18 mm cystic solid mass containing micro calcification) and finally after 5 years, the patient accepted the mass surgery without FNA. The cytopathology report revealed the papillary carcinoma of the thyroglossal duct cyst, so total thyroidectomy was down after 2 months. Pathology of right and left lobe was multinodular goiter** (**figure 3).

The patient did not receive radioactive iodine and she was advised to consume the levothyroxine tablet. After two years, she is well with 800 µg/week levothyroxine therapy without recurrence. The patient did not receive radioactive iodine and she was advised to consume the levothyroxine tablet. After two years, she is well with 800 µg/week levothyroxine therapy without recurrence.Clinical and histologic characteristic of patients were shown in table 1.

**Figure 4:**
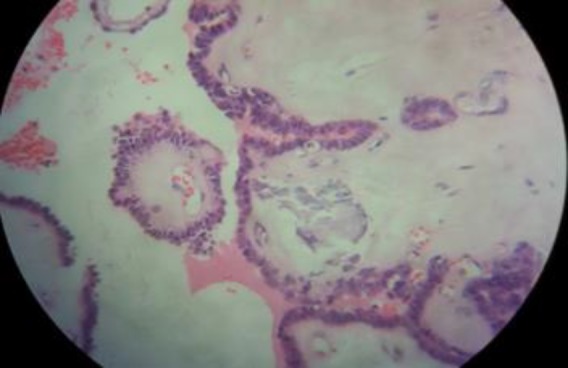
Papillary microcarcinoma arising from thyroglossal duct cyst

**Case four: **A 32-year old woman was referred for evaluation of neck mass. History of thyroid dysfunction or neck radiation was negative and her thyroid function test was normal. Physical examination showed a soft, non-tender mass in midline of the neck at the level of hyoid bone. Ultrasonography was requested that revealed a 22×10 mm cystic structure in the neck midline and also a 7 mm hypoechoic nodule with irregular border and taller than wide shape in the thyroid left lobe. Sono guided FNA was done for thyroid nodule which was suspicious for papillary carcinoma in cytology report. The patient underwent total thyroidectomy in addition to Sistrunk procedure for thyroglossal duct cyst. In pathology report, the thyroid nodule was papillary micro carcinoma (6mm in diameter) without capsular, perineural and blood vessel invasion (figure 4). 

The thyroglossal duct cyst had no tumoral involvement. Two out of three dissected neck lymph nodes were involved (0.2 and 0.3 mm in diameter). Now, after 3 months of operation, she in on 850 µg/week levothyroxine and neck ultrasonography did not show any residual or recurrence and lymphadenopathy and laboratory tests (suppressed thyroglobulin and antithyroglobulin) are acceptable.

**Table1:** Clinical and histologic characteristic of patients

**Patient **	**Sex**	**Age**	**TGDC Ca size (mm)**	**Interval to** **thyroidectomy**	**Number of** **operations**	**Thyroid Ca size (mm)**	**Lymph nodes** **metastases**	**RAI** **(mCi)**	**Follow-up** **(years)**	**Last TG stimulated** (ng/ml)*
1	M	31	NL	3 m	1	0.9	NO	NO	7y	0.9** no stimulated
2	F	29	30	6y	1	NL	NO	100	2y	0.26
3	F	43	28*20	5 y	2	NL	NO	NO	2y	0**no stimulated
4	F	32	NL	2 m	1	0.6	Yes	No	3m	2.4

ΤGDC: thyroglossal duct cyst, Ca: carcinoma, RAI: radioactive iodine, NL: normal *on stopping thyroxine

**No stimulated (this patient procedure was near total thyroidectomy)

## Discussion

The thyroid gland moves from the initial area in the foramen cecum to its final position below the thyroid cartilage trough the thyroglossal tract. The thyroglossal duct usually atrophies and closes before birth .Incomplete atrophy of the thyroglossal tract creates the basis for the origin of a thyroglossal duct cyst (TGDC) which is usually benign. A thyroglossal remnant may be present as a cyst, a fistula with or without an ectopic thyroid tissue. (17, 18).

TGDC carcinoma is extremely rare and most of them are papillary carcinoma. Usually, the malignancy diagnosis is made after the cyst excision. There are two theories regarding the TGDC carcinoma origin: the first theory supports the TGDC origin from cancer, while the second suggests a metastasis from a thyroid cancer (19). As a result, there is no consensus about concurrent total thyroidectomy and thyroglosal duct cyst in papillary carcinoma cases,(20) although some authors do not recommend this procedure on a routine basis(21, 22).

A rational approach to the TGDC cancer must consider both the intrinsic tumor characteristics and the extension of the disease. Most of the time, it is impossible to distinguish the malignant TGDC from the benign cyst before surgery. Malignancy should be suspected if the thyroglossal duct cyst is hard, irregular, fixed, rapidly growing, and associated with palpable neck lymph nodes (23, 24). It is believed that some information are in favor of a primary TGDC cancer with secondary extension to the thyroid compared to primary thyroid cancer: 1. younger age (nearly a decade younger), 2. larger tumor size, 3. more frequent extension to adjacent soft tissues, 4. higher lymph node involvement (the central compartment less frequently involved) (25). Two patients in our report had TGDC carcinoma without thyroid cancer and two cases had thyroid cancer without TGDC involvement. The age of patients was not significantly different. One of the patients had lymph node involvement. Tumor size was higher in patients with primary TGDC carcinoma compared to patients with thyroid cancer (30 and 40 mm vs. 9mm). All of our cases underwent concomitant total thyroidectomy and TGDC excision. One of the patients received the radioactive iodine. None of them had recurrence.

Ultra sonography (US) and fine needle aspiration cytology (FNAC) of the cyst may help diagnose the TGDC cancer preoperatively (21) .The solid mass within the cyst and/or regional calcifications suggest papillary carcinoma. However, TGDC cancer may exist even with a normal US and FNAC. Ultrasonography of our two TGDC cancer cases showed the solid calcified component in TGDC in our first case and a cystic solid mass containing micro calcification in another case. The mass FNA of first case was in favor of papillary carcinoma and another case refused the FNA. One of the cases had ill-defined lymphadenopathy in level 1B but its pathologic evaluation was normal.

The optimal management of TGDC cancer is still controversial. In the study by de Tristan et al., TGDC cancer was found to be present in only 1.4% (4 out of 352) of TGDC, and all of them were papillary carcinoma. Three of the 4 patients underwent total thyroidectomy (TT) but none were found to have a second carcinoma in the thyroid. They did not recommend total thyroidectomy, along with thyroglossal duct cyst surgery(25) . In this regard, some authors suggest surgical decision making based on patient risk stratification, it is recommended that Sistrunk procedure (SP) alone be performed in low risk situations with a clinical and radio logically normal thyroid gland.(21, 24). The low risk situation is defined as age < 45 years, tumor size < 4 cm, no prior head and neck radiation exposure, no soft tissue invasion, no distant or lymphatic metastasis, and no aggressive tumor histology (24, 26). The addition of total thyroidectomy and radioactive iodine ablation (RAI) is done in high risk patients and in cases with positive surgical margins (21). All of our cases had lower than 45 year, tumor size <4 cm, without history of head and neck radiation and without soft tissue or distant or lymphatic metastasis, none of them had aggressive tumor histology. Similar to Tristan et al study, the thyroid lobes in our cases with TGDC cancer were not involved. 

In conclusion TGDC can become malignant. TGDC cancer is rare and is usually diagnosed postoperatively, but ultrasonography report and fine needle aspiration cytology (FNAC) of the TGDC mass can be helpful. The best TGDC cancer approach is a controversy. According to the previous reports and also our case reports, it seems that in TGDC cancer patients with normal thyroid ultrasonography, the thyroidectomy may be not necessary. However, more detailed studies are needed to make the best decision.
